# The social practice of rescue: the safety implications of acute illness trajectories and patient categorisation in medical and maternity settings

**DOI:** 10.1111/1467-9566.12339

**Published:** 2015-09-18

**Authors:** Nicola Mackintosh, Jane Sandall

**Affiliations:** ^1^ Division of Women's HealthKing's College LondonLondonUK

**Keywords:** ethnography, hospitals, maternity services, safety, risk, quality of care

## Abstract

The normative position in acute hospital care when a patient is seriously ill is to resuscitate and rescue. However, a number of UK and international reports have highlighted problems with the lack of timely recognition, treatment and referral of patients whose condition is deteriorating while being cared for on hospital wards. This article explores the social practice of rescue, and the structural and cultural influences that guide the categorisation and ordering of acutely ill patients in different hospital settings. We draw on Strauss *et al*.'s notion of the patient trajectory and link this with the impact of categorisation practices, thus extending insights beyond those gained from emergency department triage to care management processes further downstream on the hospital ward. Using ethnographic data collected from medical wards and maternity care settings in two UK inner city hospitals, we explore how differences in population, cultural norms, categorisation work and trajectories of clinical deterioration interlink and influence patient safety. An analysis of the variation in findings between care settings and patient groups enables us to consider socio‐political influences and the specifics of how staff manage trade‐offs linked to the enactment of core values such as safety and equity in practice.

## Introduction

In line with the modern ordering of social phenomena (Bayatrizi 2008: 51), the acutely ill patient has become ‘an object of interest, inquiry, demystification, quantification, surveillance and regulation’. In this article we seek to extend the body of literature on patient eligibility, categorisation and disposal (that is, the next stage of intervention, treatment or transfer) beyond the emergency department to maternity and the general medical ward. We explore the social practice of rescue, in particular the sociocultural influences that guide the categorisation and ordering of acutely ill patients in these different hospital settings. The article's novelty lies in its consideration of meso‐level and micro‐level ordering practices in conjunction with patients’ acute illness trajectories. Our investigation adds to the sociological literature on both categorisation and illness trajectories. It also shifts conceptualisations of managing acute illness beyond bounded individual and team features and instrumental notions of safety solutions, to acknowledge in addition links with wider socio‐political influences, including the management of institutional risk.

## A focus on rescue in the context of acute illness

Providing an effective safety net for patients in general hospital wards involves the surveillance and timely or appropriate management of patients whose conditions may vary from stable to acutely unwell. The onset of critical illness appears to be often predictable (Schein *et al*. 1990). More effective rescue at an earlier stage is likely to lead to both health and economic gains by reducing cardiac arrests, intensive care unit (ICU) admissions and mortality rates (Buist *et al*. 1999, Hodgetts *et al*. 2002). This includes the timely management of severe maternal morbidity (for example, sepsis, post‐partum haemorrhage and pre‐eclampsia). In this article we focus on critical illness in the high stake settings of general medicine and maternity. Both specialties are undergoing rapid innovation in service delivery, and are characterised by changes in patients’ characteristics, workforce roles and responsibilities and shifting interfaces with other services (for example, primary care, emergency departments and critical care).

Ward profiles in acute care have become more complex, resulting in increased patient acuity and a higher workload as a result of an increase in healthcare technology and the need to care for an ageing population with multiple comorbidities (Bion and Heffner 2004). In this environment, opportunities for rescue are often missed. There is widespread evidence of ward staff's failure to recognise warning signs of deterioration and difficulties in interpreting and instituting appropriate clinical management once concerns have been identified (National Confidential Enquiry into Patient Outcome and Death [NCEPOD] 2005, National Patient Safety Agency 2007).

Maternity services are also under strain, partly as a consequence of an increasing birth rate, and a population of mothers that is generally older than they used to be, with some groups experiencing more comorbidities (National Audit Office 2013, Royal College of Midwives 2013). The volume of births to women born outside the UK has also risen; these mothers often have more complicated pregnancies, have more serious underlying medical conditions or may be in poorer general health than women born in the UK (Centre for Maternal and Child Enquiries [CMACE] 2011). While for the most part, pregnancy and birth are normal physiological processes, emergencies can develop rapidly and unexpectedly. Delayed detection of severe illness in women before, during and after childbirth can lead to poor outcomes for women and their babies (CMACE 2011).

Current risk management strategies in maternity and acute care settings include a plethora of safety systems and tool kits, such as vital sign charts and care protocols to standardise escalation pathways (King's Fund 2012, National Institute of Health and Clinical Excellence [NICE] 2007), track and trigger tools and decision aids to shape the interpretation of clinical deterioration and appropriate intervention (CMACE 2011, NICE 2007); communication protocols to provide junior staff with the mandate to ask for help (King's Fund 2012, NICE 2007); individual and team training to improve ward staff skills in recognition and response (Smith *et al*. 2002) and outreach or medical emergency teams to provide access to specialist support (Winters *et al*. 2013). Despite these, management of acute illness remains problematic (CMACE 2011, NCEPOD 2012).

We suggest that widening the analytical lens beyond individual and team processes and technical safety solutions may enhance our understanding of why management of the acutely ill represents such a ‘wicked problem’. Wicked problems are those that are difficult or impossible to solve on account of their contradictory nature and associated interdependencies, which mean that solving one aspect may lead to fresh problems (Rittel and Weber 1973). Drawing on sociological concepts of patient trajectories and categorisation enables us to situate rescue practice in the wider social practice of hospital care, and thereby usefully examine associated context, congruencies, complexities and tensions.

## Managing patients’ trajectories in hospital settings

Strauss *et al.’*s (1985) notion of the patient trajectory offers a useful theoretical resource for the study of rescue, since it encompasses not only the unfolding pathology of the patient condition but also the organisational work undertaken to accomplish that trajectory, and its consequences for the relationships between those involved. They suggest that technological specialisation and the complex bureaucracy of healthcare have resulted in fragmentation of care processes. They identify two characteristic features to healthcare work. Firstly, unexpected contingencies arise from both patients’ disease processes and a whole range of organisational and technological sources. Secondly, patients react to and affect healthcare work. These combined features create the potential for trajectories of care to be complex and highly problematic.

Strauss *et al*. (1997) observe that the extent to which various dangers and risks associated with the trajectory phases can be mapped out determines the coordination of the total arc of work, including the bundles of implicated tasks. They distinguish between trajectories where an illness is well understood, and which are amenable to control by standardised policies, and highly problematic trajectories that involve attendant uncertainties:

These uncertainties greatly increase the probability and even cumulative impact of certain kinds of mistakes stemming from the tasks themselves, from possible consequences of doing the tasks and even from the organisation of error work itself, which can lead to unanticipated new mistakes (Strauss *et al*. 1997: 243).

Various authors have usefully drawn on Strauss’ writings to progress understanding of the linkages between individual trajectories of care and broader health and social care systems (Allen *et al*. 2004, Mesman 2008). We extend the use of Strauss *et al*.'s work to include the hospital ward setting (including the maternity unit), and to situate the coordination of patient trajectories of clinical deterioration with other types of clinical work, ward cultures and temporal‐spatial influences.

## Categorisation and ordering practices

The sociological literature on ordering practices offers an additional framework to examine sense‐making regarding acutely ill patients. Roth (2005) notes that while ordering practices are ordinarily unproblematic and invisible, ‘the concrete world and the processes of differentiation are always situated, local, and saturated with contingencies’ (Roth 2005: 584). Roth suggests that there are ‘few empirical studies of the real‐time production, use and impact of classification in everyday work praxis’ (2005: 15). Bowker and Star (1999) agree: ‘what is missing is a sense of the landscape of [classification] work as experienced by those within it’ (p.65). In this article we attempt to address this gap by articulating the dynamics of the landscape of acute illness in medical and maternity settings.

Calculations around prognosis and acuity differ inter‐professionally; variance depends to some extent on the different criteria used (Degner and Beaton 1987). Provider rationing on the basis of the candidacy, or eligibility (Dixon‐Woods *et al*. 2006) of patients who are considered more or less worthy of care determines the way that particular patients gain access to services (Nugus and Braithwaite 2010). Clinical, social and organisational categorisation work occurs at the point of initial access to hospital care (Dingwall and Murray 1983, Dodier and Camus 1998, Hughes 1980, Jeffery 1979, Vassy 2001). Our interest extends this lens to legitimacy work that takes place in the acute hospital structure. Using this literature in conjunction with theories on patient trajectories enables us to explore meso‐level and micro‐level distinction practices separating acute illness from other categories, and the consequences of this and other sociocultural factors for patients’ trajectories.

## The research project

Within the context of a national policy imperative on the poor management of deterioration in clinical practice, this research was part of a larger programme of National Institute for Health Research (NIHR)‐funded work exploring how services are organised to improve patients’ journeys through and across care systems. This study aimed to explore how the deterioration and escalation of care are understood in the workplace, and the intended and unintended consequences of safety strategies and tools introduced to facilitate management of acutely ill patients. The study design involved an ethnographic approach over a 2‐year period (2009–2010) and was organised into two phases. Data were collected in the medical directorates (> 150 hours of observations, January to December 2009), and then in maternity services (> 120 hours of observations, February to August 2010). Findings on the role of safety systems in medicine (Mackintosh *et al*. 2011) and maternity (Mackintosh *et al*. 2013) have been reported. Findings from patients’ (30) and relatives’ (11) perceptions about their role in contributing to the escalation of care have also been reported (Rainey *et al*. 2015, Rance *et al*. 2013). This article focuses on the social structuring practices that shape patients’ trajectories and draws on staff interviews, observation and documentary data from both maternity and medicine. The analysis of the variation in findings between the two care settings provides insights into relationships between meso‐level and micro‐level systems.

The ethnographic approach to the study of medical work offered an effective means of uncovering how rescue is accomplished, and the relationships between this work and other structural factors. Ethnographies of work practices reveal ‘the unacknowledged, the hidden, the insider knowledge, the unwritten but pervasive rules governing jobs’ (Smith 2007: 222). The research was based across two urban inner city hospital trusts. The pseudonyms, Eastward and Westward, maintain the anonymity of the sites. Each Trust's medical directorate consisted of ten wards, altogether admitting 15,000—20,000 patients per year. Each had a maternity service providing care for around 6000 women annually. Eastward had an obstetric unit (mixed high and low risk care environment) while Westward had both an alongside midwifery unit (providing care for women classed as low risk) and an obstetric unit (a predominantly high risk environment). Both trusts provided services for home birth and cared for women who were transferred in from home. Table 1 details structural aspects of the sites. Both organisations were similar in terms of location, population served, admission rates and ward settings.

**Table 1:** Structural features of the two research sites

Contextual features	Westward	Eastward
Hospital descriptor	UK acute teaching hospital	UK acute teaching hospital
Location	Inner city	Inner city
Demographics	Mobile population, high ethnic diversity	Mobile population, high ethnic diversity
General medical service	15,000–20,000 patients admitted per year 10 general medical wards	15,000–20,000 patients admitted per year 10 general medical wards
Study ward/beds	General medicine with respiratory specialty/30 beds	General medicine with diabetes specialty/28 beds
Safety systems/tools in use across medical service	Track and trigger tool^a^ Critical care outreach team^b^	Track and trigger tool
Maternity service	> 6500 births per year Obstetric unit, alongside midwifery unit, antenatal/postnatal ward, antenatal day unit	5500 births per year. Obstetric unit, antenatal/postnatal ward, maternal assessment unit
Study unit(s)	Obstetric unit alongside midwifery unit	Obstetric unit
Safety systems/tools in use across maternity services	Partogram^c^ Track and trigger tool Critical care outreach team	Partogram Track and trigger tool

a A set of rules to distinguish when vital signs become of concern and appropriate trigger actions that need to be taken as a result.

b A team set up to facilitate management of acutely ill patients on the ward.

c A chart to plot the progress of labour to allow for early detection of problems with both mother and baby.

After ethical and research governance approval was obtained (08/H0808/178), data were collected by the first author (who has a critical care nursing and social science background) in the medical directorates, and by the first author and two additional researchers (one with a midwifery and one with a sociology background) in maternity services. While the selection of a particular field is directed by a theoretical interest in a specific problem, the significance of the many events contextualising the problem can never be determined a priori. Rather than focusing solely on emergency episodes when patients became acutely unwell, we learned about the everyday reality and routines of each area and collected data on space, actors, activities and objects, as well as events and time goals (Robson 1993).

Observation of ward or unit activity and medical work enabled an additional opportunistic focus on events and interactions as they arose. In addition to the unstructured observation of ward and unit work, we shadowed a cross‐section of medical staff to gain insight into their routine working day as well as their on‐call episodes (Table 2). Recruitment was done by e‐mail as well as face to face invitations (via meetings and during fieldwork on the wards). Fieldwork included the observation of management‐level meetings about the care of acutely ill patients. Jottings or headnotes were audio‐recorded immediately after the conclusion of each day's observations (Emerson *et al*. 1995) and transcribed.

**Table 2:** Data collection

** **	Westward		Eastward	
	Medical	Maternity	Medical	Maternity
Observations	10 ward shifts, 4 shifts shadowing medical staff, 8 acutely ill patient in hospital committee meetings	4 alongside midwifery unit shifts, 6 obstetric unit shifts, 11 risk meetings	8 ward shifts, 4 shifts shadowing medical staff, 1 shift with outreach team, 10 acutely ill patient in hospital committee meetings	11 obstetric unit shifts 5 risk meetings
Interviews	HCAs: 2 Ward nurses: 3 Outreach nurses: 2 Doctors: 5 Managers: 2 Total: 14	Midwives: 8 Obstetricians: 7 Anaesthetist: 2 Neonatologist: 0 Managers: 6 Total: 23	HCAs: 2 Nurses: 4 Physio: 1 Doctors: 9 Managers: 5 Total: 21	Midwives: 9 Obstetricians: 4 Anaesthetist: 1 Neonatologist: 1 Managers: 6 Total: 21
Documentary review	Ward protocols, audits, minutes of acutely ill patient in hospital committee meetings, ICU admission audits	Ward protocols, audits, minutes of risk meetings	Ward protocols, audits, minutes of acutely ill patient in hospital committee meetings, ICU admission audits 7 months data from intelligent assessment technology	Ward protocols, audits, minutes of risk meetings

HCA, healthcare assistant

Interpretations of practice were followed up during in‐depth interviews with a range of staff, to test out assumptions and clarify contradictory understandings. Staff members were recruited for interview by a mixture of e‐mail and face to face contact. The interviews were conducted in private spaces (offices, meeting rooms and coffee rooms) and lasted between 20 and 70 minutes. Interviewees were purposively selected for their theoretical representativeness, in terms of the categories, substructures and networks of the social organisation (Johnson 1990).

All transcripts of observations, interviews, minutes of meetings, and documentary data were imported into N‐Vivo version 8. This facilitated content and thematic analysis. The analysis aimed to preserve particular patients’ rescue trajectories, including their care provision and interactions, over periods of fieldwork. We drew on the framework approach, which involves a series of interconnected stages that enable the researcher to move back and forth across the data until a coherent account emerges (Ritchie and Lewis 2003). This involved making sense of the core concepts, which had emerged from the refined categories and final themes, the clinical literature and the theoretical sociological perspectives.

Glaser (1998) recommends three codes of increasingly abstract categorisation: substantive, theoretical and core. Five substantive codes were generated: routine work; identification of a problem; asking for help; responding and structural influences. We developed four theoretical codes: enactment of safety work; division of labour; boundary work and socio‐technical systems. Rescue work formed the core code. The findings reported in this article draw from the structural influences code and link to all four of the theoretical codes. We consider the clinical presentation, categorisation and care of acutely ill patients, and how these relate to wider institutional sociocultural and political influences. These relationships inform comparisons between settings and enable an analysis of the work involved in managing linked processes such as efficiency, responsiveness, effectiveness and safety.

## Findings

### The rescue imperative in the context of maternity and medical care

Across the two care settings, the nature of the clinical emergency and its demands for immediate medical attention emerged as a core organising frame. The rescue imperative underpinned the logic of acute hospital care. The concept of the trajectory (Strauss *et al*. 1985) was important for setting timelines and structuring care in relation to sickness (and death) and birth (De Vries 1981). Both organisations had invested in systems and processes designed to facilitate intervention in the time period preceding the patient's collapse, potentially halting and reversing the trajectory of clinical deterioration and imposing order on the associated unpredictability and crisis. Rescue provided a social and ideological mandate for practice (Chapple 2010). The accomplishment of effective and timely management of clinical deterioration demonstrated the essential business of acute and maternity care delivery, as this consultant physician says:

We observe patients for signs of deterioration. This is the most basic aspect of hospital care. Here it's done reasonably well, at a level where if you were running a dry cleaning operation if it was much worse you'd be out of business because you'd lose so many suits. (Westward, Physician, 11)^1^

In maternity, the rationality and moral worth of urgent rescue work was particularly evident. While acute events were relatively infrequent on the medical wards, on the obstetric units the management of clinical crises was normalised as part of ‘what we do round here’. Discourse on the units frequently involved midwives and doctors recounting atrocity stories (Dingwall 1977) as a means of recognising the accomplishment of heroic rescue work:

At half past ten the emergency buzzer went off in room 5 went so the coordinator and medical staff who were in the office ran to the room. Five, ten minutes later the coordinator came back saying it was a cord prolapse, minutes later the woman was transferred to theatre with one of the doctors astride her, holding the cord up.^2^ Once the coordinator had checked all the staff were present in theatre she returned and very quickly the office was quiet again and the rest of the unit appeared to carry on as normal. (Westward, FN7)

The midwives are chatting about the TV programme, ‘One Born Every Minute’, saying how lovely and cosy that environment was, how it didn't reflect practice ‘here’. One midwife recounts an episode showing an emergency transfer of a woman in labour to theatre. She says scornfully ‘we would have done it much faster, we would have had her in theatre in seconds, not like the slow process that we saw on telly’. The other two murmur their agreement (Eastward, FN5).

While rescue served as an organising principle across all settings, the enactment of local rescue processes reflected variance in patient groups, sociocultural norms and trajectories of clinical deterioration. An important feature in maternity was the view that the ‘women aren't sick, these are women who are young, they are healthy’ (Westward, Midwife, 15). Difficulties distinguishing changes associated with normal pregnancy from the signs of acute illness made the recognition of deterioration a complex process: ‘Women are incredibly resilient, and can deal with enormous blood loss’ (Westward, Anaesthetist, 11). By the time that a woman started to show signs of physiological compromise, the underlying problem was often fairly well advanced, necessitating prompt action to avert avoidable harm for the woman or her child.

In medicine, the pattern of deterioration in physiological observations was reported to provide more of a window of opportunity for detection and intervention than in maternity. Patients could show signs of clinical deterioration for hours or even days as their acute illness developed. The logic of rescue in the acute care setting was linked to the alignment of symptoms and pathology, and the ability to halt and potentially reverse clinical deterioration. As a physician noted:

We are trying to improve the care of people that are getting sicker in the Trust, by identifying them and identifying pathways by which their condition might be improved. (Westward, Physician, 16)

### Ensuring safety, quality and efficiency

Across both settings, a discourse of prediction, control and avoidance of the accidental (Green 2003) provided a frame of reference for managing acute illness. Adverse outcomes such as cardiac arrest, a patient's unexpected admission to intensive care and an unexpected death in hospital represented the mismanagement of illness trajectories. Risk‐management techniques had been introduced as a means to engender the public's confidence in the hospital institutions. Problems with rescue, like other patient safety problems, were constructed as amenable to methodological and technical improvement (Jensen 2008):

It was shameful what went on here [in the past] because awful things happened to patients and there was no systematic quality control or control of process or whatever, it was just, well, night follows day and these things happen. Too often the patients would be allowed to deteriorate and either die or get serious complications or whatever, you know, because of failure of effective recognition and response. [We've recognised] that it's important to quality control management of acutely ill patients. (Westward, Manager, 11)

We want good outcomes for mothers and babies, therefore it follows when something goes wrong, there should be reasons that can be understood and processes put in place to try and reduce the chances of those things happening in future. (Eastward, Obstetrician, 1)

Track and trigger tools had been introduced to both medical and maternity settings to enable the detection of clinical deterioration. Track and trigger systems are simple algorithms detailing a plan of action based on vital sign measurements. Points are allotted to particular vital sign measurements on the basis of physiological derangement from a predetermined range, generating an ‘early warning score’. These systems help shape constructs of deterioration and the point of intervention, and provide legitimacy for calling for help (Mackintosh and Sandall 2010, Mackintosh *et al*. 2014). Escalation pathways had been put in place to ensure that acutely ill patients had access to appropriate critical care services.

In maternity, the added value of the track and trigger system in controlling for risk and ‘problematic’ trajectories was limited, given there was little prior warning of the event for a number of obstetric complications. Individual midwives and obstetricians were able to resist using the system routinely in the postnatal period on account of concerns that it medicalised normal childbirth trajectories. The potential gain in detecting morbidity early was also perceived to be small when offset against the increase in workload involved in managing follow‐up vital sign recordings and false positive referrals.

However, the unpredictability of maternity collapse and the high social worth of women (and their babies) reinforced a need for management and organisational control of rescue practice (Green and Armstrong 1993, Walsh 2006). As Timmermans (1999) noted in his study of resuscitation efforts, value judgements about the patient's age, quality of life and perceived seriousness of the illness render these variables social. Managing trajectories in this setting involved being prepared for a crisis and having the resource to respond quickly to changes at organisational level (unit capacity). Ensuring flow through the system to ensure access to obstetric resource for crisis management was essential for efficiency and effectiveness:

We have got two patients and our patients aren't patients, they're well and start off entirely normal and then they have life‐threatening problems which are unexpected disasters every time. This is not a geriatric ward … Care of these cases is always highly emotional. (Westward, Obstetrician, 1)

Accounting for risks was an important part of securing trust in the maternity service. All obstetric emergencies such as maternal haemorrhage, maternal or infant admission to intensive care, maternal or neonatal infection, stillbirth and maternal death were classed as clinical incidents and investigated:

Maternity is streets ahead of every other directorate in the Trust [with regards to risk management], because we have so many risks, but there's a much more transparent approach, so if an incident occurs you're likely to get three risk reports rather than nothing. (Westward, Manager, 13)

The risks posed by the poor detection of and response to acute illness were substantial in terms of poor health outcomes for mother and baby, and litigation costs incurred for remedial care and compensation:

Everybody thinks that if they get pregnant they're going to have a perfect pregnancy and delivery and a perfect baby, and if it goes wrong somebody must be to blame. (Eastward, Obstetrician, 17).

Within the medical ward setting, managing risks and trajectories presented a different set of issues. As one consultant physician explained, ‘we have many deaths and many people that are old, frail and sick and probably going to die in the next year’ (Eastward, Physician 16). Audit data provided a window to enable managers and clinical leads to distinguish between patients’ trajectories and categorise those that were problematic. Certain trajectories were less amenable to control, which had implications for the scope of quality improvement of associated rescue processes. For example, head and neck cancer was associated with sudden catastrophic haemorrhage and coronary events were associated with sudden collapse which limited the opportunity for improving detection and response behaviour:

Our ultimate goal is that there will be no cardiac arrests in ward areas … With straightforward medical patients there are usually markers [of deterioration] that are present. When we speak to cardiac teams then it's not quite so easy to predict, in the last month the two cardiac areas had the vast majority of the arrests in ward areas. I think that's to do with cardiac physiology. And the other ones that we find have quite a few arrests are head and neck cancer where the patients haemorrhage. So I've met with the nurse specialist for those patients and she's looking if there's anything that could be done. (Westward, Manager, 10)

While the default position in medical wards was to resuscitate and rescue unless a written mandate specified otherwise, something much closer to an efficacy model was evident in practice, with its emphasis on the appropriateness of resuscitation and the escalation of care orders:

Five of us are at the acutely ill patients’ meeting, including a physician (the chair), a palliative care consultant, a clinical governance lead and a critical care nurse. The chair discusses the potential for the Trust to develop three care pathways for all hospital patients: (a) those for active resuscitation (b) those who need resuscitation but ITU [therapy intensive unit] admission may not be in their best interest and (c) those who need palliative care and are not for resuscitation or escalation of care. (Eastward, Field notes 8)

Quality and efficiency frames of reference (Hillman *et al*. 2013) were used to rationalise these distinction practices on the grounds of avoidance of the distress associated with ‘misplaced rescue’. At both trusts a number of incident and audit reports of poor decision‐making around end of life care and cases of inappropriate resuscitation had contributed to a growing pressure for a change to the way that acute care was provided. At Eastward there was an additional pressure to conserve critical care resources, which were limited in comparison to those at neighbouring trusts:

[This] organisation has limited critical care capacity and has to make prioritisation decisions … Certain patient groups have a relatively high mortality when admitted to a critical care setting namely, patients in chronic renal failure, patients with HIV infection, patients with haematological malignancy, and any patient over 80 years of age. Such patients are at high risk of ‘futile’ critical care admission … Any patient [with these conditions] or over 80 years of age should automatically trigger a case conference within 48 hours between the admitting consultant and the duty critical care consultant. The case conference should also establish in writing a comprehensive treatment plan to include decisions around escalation and resuscitation. (Excerpt from Eastward critical care policy document, unpublished, 2007).

Selective access to critical care was legitimised to prevent the burden of mortality being shifted from the ward to the intensive care unit, thus diverting resources that could be used for other patients more likely to benefit from this specialist service.

Local systems of categorisation in rescue presented a simplistic picture of clinical work, creating notions of transparency (Tsoukas 1997). Certain additional risks associated with these processes largely escaped organisational attention. We turn first to the hidden problem of managing the deteriorating conditions of elderly patients with complex conditions in medical wards, and secondly, boundary distinctions between maternity rescue resources.

### The acutely ill ‘crocks’ in medical wards

Crocks (Becker 1993) are those patients who have a number of complaints but no discernible pathology and puzzles to solve. Modern healthcare is poorly designed to meet the needs of people with many things wrong at once; these patients are constructed as inappropriate for the system (Rockwood and Hubbard 2004). The inbuilt algorithms for the track and trigger systems used in acute care were designed for an average patient on a hospital ward (Suokas 2010). A number of acutely ill patients on the medical wards presented with more than one chronic condition compounding their acute illness (Tadd *et al*. 2011). Baseline readings for vital signs for elderly patients with complex, long‐term conditions often lay outside the normal range or lay close to the thresholds set for the escalation of care. Patients with chronic respiratory disease often triggered a false positive alert even when their condition was stable. The trigger system was less able to discriminate between this stable state and when patients’ condition was deteriorating:

With chronic obstructive pulmonary disease patients … I think sometimes a deterioration in their condition is a lot harder to pick up on, because they've already got low saturations, they've already got high respiratory rates. Sometimes it takes a lot longer for the score to pick up a problem. Whereas someone young who comes in with tuberculosis you pick it up quite quickly because there's nothing else wrong with them, so there's no reason for them to be like that. (Westward, Nurse, 3)

Two types of additional risk for these patients were introduced by the systems. Firstly, there was greater need for nurses to use clinical judgement in assessing the significance of the risk scores and alerts in the light of the patient's specific condition. This was difficult at times, given that the track and trigger systems were designed to provide jurisdictional control over a craftsman‐type model of practice (Harrison and Smith 2004). Secondly, a risk associated with persistent over‐triggering was the normalisation of high scores. A woman who had a cardiac arrest on one of the wards died later in intensive care after scoring highly for a number of days due to a raised temperature. A history of mental health problems had complicated the presentation of her illness. Over time, the team collectively became conditioned to the patient's raised score:

We had a mental health patient who was transferred from a psychiatric ward to us with a pyrexia of unknown origin, her temperature was high, had always been high, she'd always been tachycardic [a high heartbeat rate], she'd always had low blood pressure, so when you looked at her pattern it was like normal … well, normal for her, you know. Although it's been the norm, every time I did [the observations] I let somebody know that this is what [the chart] says … The doctor came and reviewed the patient again, but just said to continue with the current plan. (Eastward, Nurse, 4).

Within the medical directorate, the wards were categorised according to particular medical specialties that aligned with these organs, such as cardiology and respiratory, in addition to specialised service wards such as health and ageing, and rehabilitation wards. Patients with more than one condition (as in the example of the mental health patient) were disadvantaged by this categorisation process. These patients often fell through the gaps of the bureaucratic structure of the hospital and escaped timely attention. They became lost between specialisms with no one taking an overview of the person as a whole (Tadd *et al*. 2011):

When a patient's condition is very complex, lots of teams are involved but often nobody's really taken ownership. The patient can end up being slightly in limbo, with lots of opinions, but no action. Particularly if there is some degree of disagreement between specialties, the end result is that nobody does anything, there's a bit of a waiting game, and the patient continues to deteriorate. (Eastward, Physician, 15)

Because of elderly patients’ multiple conditions, their medical teams frequently required specialist advice from other teams (such as neurology or cardiology). Specialist teams were observed to protect the boundaries of their practice. Acceptance of a referral was contingent on evidence of sufficient diagnostic tests and the presence of new, ‘interesting’ clinical signs and symptoms. Dodier and Camus (1998) observed that interesting cases are those ‘that are difficult to solve, but where there is hope of clarification, which excludes rather poorly differentiated conditions such as alterations in the general state of elderly people’ (p. 16). The safety and quality of care was compromised for these crocks (Becker 1993):

On the ward round we see Fred who is an elderly man who collapsed at home. His medical team have sought advice from the neurology team as they are unsure if his collapse is cardiac or neurological in origin. The neurology team have declined to review Fred this admission as they have already seen him recently in the outpatient clinic. (Westward, Field notes 11)

Underpinning risk rationalities and the micro‐politics of the care of the acutely ill in medicine was a moral evaluation founded upon the application of concepts of social worth common in the larger society (Roth 1972). Rescue was organised primarily around the timely and effective treatment of patients with one specific condition (Calnan *et al*. 2013). This reflected the primacy of specialism over generalism in hospital care, and highlighted an inbuilt discrimination against the major client group for acute care, that is, older patients with multiple comorbidities.

### The primacy of the ‘real emergency’ in maternity

In maternity, the obstetric unit resource required protecting to ensure that those genuine emergencies were able to access specialist monitoring and medical treatment. Ensuring the safe crisis management of those already identified as acutely ill took priority over the safety and quality needs of women who were already housed on the obstetric units who had a potential risk of developing a complication:

The midwife co‐ordinator on the obstetric unit tells me about a recent shift she worked where she delayed accepting an admission from the antenatal ward on account of the needs of the women already housed on the obstetric unit. She had two women with twins in labour, all the labouring rooms were occupied and the waiting room was full of women waiting to be assessed. She said she perceived the unit was operating at ‘maximum safety levels’. The woman who was waiting for the transfer had already been identified as unwell; while on the ward, her condition deteriorated quickly, she was found to be septic and needed a crash [emergency] caesarean section to deliver her baby. The midwife explains that she has been asked to write a statement to account for her decision. (Eastward, Field notes 6)

While the primacy of rescue enabled a timely response to the sudden obstetric emergency, detection of the slow drift of clinical deterioration in the antenatal and postnatal period was harder to manage. Boundary distinctions between the need for midwifery or obstetric‐led care, and physiological and pathological trajectories were difficult to negotiate at times. This was particularly evident at Westward, where postnatally, women were often housed on the alongside midwifery unit as overspills from either the obstetric unit or postnatal ward. Medical staff tended to cross the boundary between the obstetric and alongside units only when an emergency call was put out. Securing a medical response for a woman whose condition was deteriorating slowly on the alongside unit was contingent on the presence of new symptoms which established a woman's’ legitimacy for medical attention as a ‘real emergency’ (Dodier and Camus 1998):

If you pull the emergency button immediately you have both the senior midwives from the obstetric unit or the doctors … and emergencies are acted on very quickly. But if you have someone who's a postnatal outlier needing to be reviewed, it may take a few hours before that woman gets reviewed … that kind of obstetric support is not here because it's set up for down in the postnatal ward. (Westward, Midwife, 4)

We had a woman who had had a previous caesarean section, who had been having abdominal pains, not labour pains, generally feeling unwell and … we'd been waiting for her to be reviewed for a long, long time. In the end we decided to do a pre‐eclampsia screen, it came back and her bloods were very abnormal. As soon as we had the proof of the bloods she was round next door as quick as a flash, but we'd been waiting eight, nine hours for a review. We'd been asking and escalating it [requesting a review and a step up in care]. (Westward, Midwife, 10)

Maternity services are structured around risk and women's trajectories to deal with possible crisis events. Boundaries are instituted through distinction practices. Gaining legitimate access to obstetric resources was difficult at times, particularly for women who were located outside the usual pathway through services and presented with a slow drift of deterioration, but were not yet at the point of collapse.

## Discussion

In this article we suggest that Strauss *et al.’*s (1985) study of the social organisation of medical work offers a useful framework to theorise the complex care management of rescue, which remains relatively underdeveloped (Latimer 2014, Parker *et al*. 2000, Reiger 2011). We extend our lens of enquiry beyond the critical event of a patient's collapse to decision‐making upstream. This enables us to explicate the linkages between such events and trajectories of care (Allen *et al*. 2004). Our focus on trajectories illuminates the physiological process of birth and the unfolding pathology of illness (and death). This frame provides a means for us to link the agency of those involved in organising the care of acutely ill patients with the wider socio‐political factors beyond the clinic, such as governmentality and risk (Heyman 2010, Waring 2007), death brokering (Timmermans 2005) and the medicalisation of birth and death (De Vries 1981).

Since Jeffery's (1979) dichotomy of categories of good or interesting patients and ‘normal rubbish’ was criticised for being over‐simplistic, research has tended to focus on the fluidity and interactional character of typifications in the emergency department (Dodier and Camus 1998, Hillman 2013, Hughes 1980, Nugus and Braithwaite 2010). While it is important to avoid overestimating the stability and influence of organisational bureaucratic structures and boundaries (Davies 2003), a sociology of practice needs to acknowledge the influence of organisational context, the structural elements, policies and relationships that are ‘reestablished and reconstituted through work practices’ (Timmermans 2006: 29). This article extends existing knowledge of categorisation practices in the emergency department to consider how patients’ acute illness trajectories link to the ordering processes that occur in the socially structured conditions of the acute hospital ward. The article's novelty lies in its presentation of ethnographic material that illustrates relationships between the ideology of rescue, the perceived moral worth of patients, the handling of uncertainty in patients’ acute illness trajectories and the intended and unintended consequences of risk technologies introduced to govern these trajectories for patient care.

While other studies investigating care in medical and maternity settings have highlighted how the labelling of patients adversely influences the way in which their care is organised, this article adds an important patient safety lens to the analysis. It adds to research showing how the management of risk and safety is politicised and applied locally (Annandale 1996, Brown and Calnan 2010, Scamell 2011, Tadd *et al*. 2011). Its specific contribution lies in insights generated over the often competing sets of medical, sociocultural, economic and political rationalities that face staff ‘doing’ patient safety. Reconciling quality, safety and efficiency is often difficult and requires constant trade‐offs (Dixon‐Woods *et al*. 2009, Nugus and Braithwaite 2010).

Not surprisingly, given recent policy emphasis on reducing the rates of avoidable death, which draw on cultural distinctions between the orderly and disorderly death (Bauman 1992, Bayatrizi 2008, Timmermans 2005), the rescue imperative provides a dominant organising frame across acute care. The logic of efficiency, effectiveness and safety in healthcare insists that cardiac arrests, an unexpected admission to ICU or death ought to be prevented. Risk technologies and audit data bring a degree of measurability to the management of critical illness, and help shape the organisation's generalisable knowledge about rescue work (Power 1997). An institutional audit culture that held practitioners accountable for their rescue work was particularly noticeable in maternity. Financial risk associated with failure to rescue overshadowed other risks (National Health Service Litigation Authority 2012). The management of rapidly unfolding clinical emergencies was arguably more tightly coupled to the outcome and the individuals involved in the rescue in maternity rather than in the medical settings, where clinical deterioration tended to occur over a longer period. In medical settings blame could be more easily diffused or relocated (Dixon‐Woods *et al*. 2009).

Timmermans (1999) noted that the most outstanding social characteristics are the patient's age and the perceived seriousness of the illness with regard to the resuscitative effort. We note the significance of the elderly patient with complex conditions and the urgent maternal collapse in the broader construct of rescue trajectories. Our data support previous research suggesting that the delivery of acute hospital care is poorly designed to meet the safety needs of elderly patients with complex chronic conditions (Hillman *et al*. 2013, Latimer 2014, Tadd *et al*. 2011) and gate‐keeping and legitimising practices in the maternity service contribute to problems accessing medical help (Kirkup 2015, McCourt *et al*. 2011). Our data add to the existing literature on the adverse consequences that can occur in the hospital for those who are categorised as not real emergencies (Dodier and Camus 1998) that is, ‘neither in crisis nor completely stable’ (Chapple 2010: 56), and the safety of outliers (Goulding *et al*. 2012). It extends the lens upstream from the construct of institutional death brokering (Timmermans 2005) to the active management of the physiological aspects of acute illness. Adverse events in patient safety are often not primarily due to human error at the ward level but are ‘rather a systemic – or networked – consequence of the ways in which health work is related to cultures of management, governance and science’(Jensen 2008: 322).

## Notes

1 Number in brackets in the extracts refer to transcript discriptors.

2 In this obstetric emergency, the umbilical cord had been delivered prior to the baby so the first‐line response necessitated taking pressure off the cord.
